# Genomic Epidemiology of ESBL- and Carbapenemase-Producing Enterobacterales in a Spanish Hospital: Exploring the Clinical–Environmental Interface

**DOI:** 10.3390/microorganisms13081854

**Published:** 2025-08-08

**Authors:** Sandra A. Martínez-Álvarez, María Ángeles Asencio-Egea, María Huertas-Vaquero, Teresa Cardona-Cabrera, Myriam Zarazaga, Ursula Höfle, Carmen Torres

**Affiliations:** 1 Area of Biochemistry and Molecular Biology, One Health-UR Research Group, Faculty of Science and Technology, Universidad de La Rioja, Madre de Dios 53, 26006 Logroño, Spain; sandra.martinezal@unirioja.es (S.A.M.-Á.); myriam.zarazaga@unirioja.es (M.Z.); 2 Hospital Universitario La Mancha Centro, 13600 Alcázar de San Juan, Spain; marian_asencio@yahoo.es (M.Á.A.-E.); mahuva77@yahoo.es (M.H.-V.); 3 Health and Biotechnology (SaBio) Research Group, Institute for Game and Wildlife Research (CSIC-UCLM-JCCM), 13005 Ciudad Real, Spain; greentere@hotmail.com (T.C.-C.); ursula.hofle@uclm.es (U.H.)

**Keywords:** *Escherichia coli*, *Klebsiella pneumoniae*, carbapenemases, ESBLs, OXA-48, CTX-M-15, One Health, clinical-environmental interface

## Abstract

Antimicrobial resistance (AMR), particularly due to extended-spectrum β-lactamases (ESBLs) and carbapenemases (CPs), poses a critical threat to global health. This study aimed to characterize the molecular epidemiology, resistance profiles, and genomic features of ESBL- and CP-producing *Escherichia coli* and *Klebsiella pneumonaie* (ESBL/CP-Ec/Kp) isolates from a Spanish hospital (2020–2024) and explore links to environmental reservoirs like white storks foraging at a nearby landfill. A total of 121 clinical Ec/Kp isolates (55 ESBL-Ec, 1 CP-Ec, 35 ESBL-Kp, 17 CP-Kp, 13 ESBL+CP-Kp) underwent phenotypic testing, PCR, and whole-genome sequencing (WGS). Analyses included phylogenomics (cgMLST), detection of AMR genes, plasmid typing, and comparative genomics. Among ESBL-Ec, *bla*_CTX-M-15_ was the most prevalent (60.0%), and one CP-Ec carrying *bla*_NDM-5_ was identified. WGS of 44 selected ESBL/CP-Ec isolates revealed a variety of AMR genes, and 56.8% of isolates carried class one integrons (56.8%). IncF-type plasmids predominated, and 84.1% of isolates were assigned as ExPEC/UPEC. The lineage ST131 dominated (75%), with IncF-*bla*_CTX-M-15_-carrying plasmids. Among the 18 ESBL/CP-Kp isolates sequenced, the lineage ST307 was the most frequent (44.4%), followed by ST15 and ST11, carrying a diversity of AMR determinants and plasmids (IncFIB(K), IncL, ColpVC). Virulence included *ybt* loci in ICEKp; hypervirulence genes were absent. Genomic analysis of 62 clinical isolates (44 Ec, 18 Kp) showed close phylogenetic links to stork-derived strains, with ST131-Ec and ST307-Kp from humans and birds differing just by ≤22 and ≤10 ADs, respectively, with a conserved plasmid content (i.e., IncL-*bla*_OXA-48_, IncFIB(K)-*bla*_CTX-M-15_). High-risk ESBL/CP-Ec/Kp clones persist across clinical and environmental contexts. WGS-based surveillance is key for understanding AMR spread and guiding interventions. Results support a One Health approach to combat AMR through cross-sector collaboration.

## 1. Introduction

Antimicrobial resistance (AMR) is one of the most pressing global health challenges of the 21st century. The increasing prevalence of multidrug-resistant (MDR) bacteria has led to severe limitations in available treatment options, prolonged hospital stays, increased healthcare costs, and higher mortality rates [1]. Among the most concerning resistance mechanisms are extended-spectrum β-lactamases (ESBLs) and carbapenemases (CPs), which render many β-lactam antibiotics ineffective. These resistance determinants are particularly problematic in healthcare settings, where they contribute to nosocomial outbreaks and complicate infection control measures [2,3]. Spain has witnessed a growing burden of ESBL- and CP-producing Enterobacterales (CPE), with a steady increase in prevalence over the past two decades [4,5,6,7,8]. The CARB-ES-19 study demonstrated widespread dissemination of carbapenemase-producing *Klebsiella pneumoniae* (Kp) and *Escherichia coli* (Ec) across Spain, with ST307/OXA-48 and ST512/KPC-3 as dominant high-risk clones [6]. The CARBA-MAP project further highlighted regional variation and the emergence of novel carbapenemase producers between 2014 and 2018 [7]. Complementing these findings, recent data from a tertiary hospital in Madrid revealed co-production of multiple CPs in Enterobacterales isolates, reflecting an increasingly complex resistance landscape [8]. For ESBL-producing Ec, the ITUBRAS-2 study reported a notable rise in CTX-M-15-ST131 and emergence of CTX-M-27-ST131 in bacteremia of urinary origin, underscoring their clinical relevance due to enhanced virulence and persistence [9]. These pathogens are frequently implicated in UTIs, sepsis, and ventilator-associated pneumonia [6,7], and they are increasingly detected in community and environmental settings as well [8,9,10,11,12]. CPEs also represent a critical concern in Spanish healthcare institutions. According to the European Centre for Disease Prevention and Control (ECDC), Spain is classified as a country with a high CPE incidence, though with notable regional variability [13]. To mitigate the spread of MDR pathogens, Spain has adopted several coordinated strategies: (i) antimicrobial stewardship programs (PROA) that promote rational antibiotic use and have helped reduce the consumption of carbapenems and third-generation cephalosporins [14]; (ii) infection prevention and control (IPC) measures, such as hand hygiene, contact precautions, and environmental cleaning, essential in healthcare settings [15]; and (iii) the National Plan against Antibiotic Resistance (PRAN), which supports active surveillance to detect and contain MDR infections [16]. In recent years, whole-genome sequencing (WGS) has emerged as a transformative tool in AMR surveillance. Traditional microbiological and molecular techniques remain valuable, but WGS allows high-resolution characterization of AMR determinants, plasmids, and clonal lineages [17]. Its integration into clinical microbiology enhances the ability to (i) track resistance evolution; (ii) interpret AMR through a One Health lens, linking clinical, animal, and environmental sources; (iii) conduct precise outbreak investigations; and (iv) guide individualized antimicrobial therapy [17,18].

Environmental transformation, climate change, and other anthropogenic pressures have led many wild bird species such as white storks to modify their foraging behavior. In some populations, solid urban waste from landfills has become a primary food source. This anthropization increases the likelihood of birds acquiring human-associated pathogenic and antimicrobial-resistant bacteria [19,20,21]. Several studies have reported the presence of multidrug-resistant bacteria harboring clinically relevant resistance genes in wildlife, particularly migratory birds such as the white storks [22,23,24,25]. Storks have been shown to connect landfills to a variety of other habitats and can travel thousands of kilometers during migration and thus could potentially have an important role in the dissemination of pathogens and AMR [26]. In contrast, as they are not exposed to antimicrobial treatments in the wild, their AMR burden reflects environmental contamination driven by human activity, making them valuable environmental sentinels for AMR [27,28,29]. These findings reinforce the importance of conducting epidemiological studies beyond the clinical setting.

In a previous study conducted by our group, we reported a high prevalence of ESBL/CP-Ec/Kp in fecal samples of white storks (*Ciconia ciconia*) foraging at two landfills in central Spain [30], with the detection of a wide diversity of ESBL- and CP-encoding genes among these isolates. The proximity (~4 km) of one of these landfills to a regional hospital raised concerns regarding indirect transmission mediated by wildlife. A genomic comparison was performed between Kp-ST512-*bla*_KPC-3_ isolates of storks and of clinical origin recovered in the regional hospital, detecting clonal relationships among them [30]. For this reason, in the present study, we use whole-genome sequencing to characterize all the ESBL/CP-Ec/Kp isolates recovered in the targeted regional hospital during 2020–2024 (in addition to the Kp-ST512-*bla*_KPC-3-like_ previously indicated) and to perform a new comparative genomic analysis with the stork isolates, encompassing a broader spectrum of resistance determinants, including other CP (i.e., OXA-48, VIM, NDM) and ESBL genes circulating in the hospital, with the aim to explore potential links between clinical and environmental reservoirs. In addition, we contextualize our findings within a One Health approach, exploring how AMR determinants and high-risk bacterial clones can cross ecological boundaries, ultimately informing integrated surveillance and control strategies.

## 2. Materials and Methods

One hundred and twenty-one ESBL/CP-Ec/Kp clinical isolates (55 ESBL-Ec, 1 CP-Ec, 35 ESBL-Kp, 17 CP-Kp, and 13 ESBL+CP-Kp) were obtained during January 2020 and June 2024 from the Hospital Universitario La Mancha Centro (one isolate per patient), located 4 km away from the municipal landfill (Alcázar de San Juan, Ciudad Real, Spain), and were included in this study (Table 1 and Table S1).

### 2.1. Antimicrobial Susceptibility Testing and Molecular Characterization

Bacterial identification and antimicrobial susceptibility testing were performed using the Vitek2^®^ and Vitek-MS^®^ systems (version 3.2-IVD) and Etest gradient diffusion strips (BioMérieux, Marcy-l’Étoile, France), following EUCAST guidelines. In cases where reduced susceptibility to any carbapenem was detected, carbapenemase production was assessed using the β-Carba test (Bio-Rad Laboratories, Hercules, CA, USA). For positive cases, phenotypic characterization was extended using synergy testing with meropenem and phenylboronic acid, cloxacillin, dipicolinic acid, and temocillin susceptibility (Rosco Diagnostics, Taastrup, Denmark). Some isolates were recovered from rectal swabs obtained as part of routine hospital screening for intestinal colonization in high-risk patients. These samples were plated on ChromID Carba Smart agar (BioMérieux, Marcy-l’Étoile, France) according to local infection prevention protocols. Colonizing isolates were analyzed together with clinical strains for genotypic characterization and comparative genomic analysis. All ESBL/CP-Ec/Kp isolates were sent to the Antibiotic Resistance Laboratory at the University of La Rioja for genotypic characterization of *bla*_ESBL_ and *bla*_CP_ resistance determinants (PCR and sequencing) followed by bioinformatic analysis. A preliminary MLVA (Multiple-Locus Variable-number tandem-repeat Analysis) was applied to all isolates to define clonal clusters, and one representative strain from each cluster was selected for whole-genome sequencing (WGS) (Table 1).

### 2.2. Sequencing and Bioinformatic Analysis of the ESBL/CP-Ec/Kp Genomes

The genomes of 13 *bla*_KPC-3-like_ isolates were previously reported in our previous study [30]. We obtained the genomes of 62 additional ESBL/CP-Ec/Kp isolates through WGS.

Genomic DNA from 44 ESBL/CP-Ec and 18 ESBL/CP-Kp clinical strains was extracted using the QIAmp DNA Mini Kit (Qiagen, Hilden, Germany). Subsequently, libraries were prepared with the TruSeq DNA PCR-free Sample Preparation Kit (Illumina, San Diego, CA, USA), and 150 bp paired-end reads were sequenced in a NovaSeq 6000 System (Health in Code Facility, Valencia, Spain). Illumina reads were quality-checked with FastQC v0.74 software (https://www.bioinformatics.babraham.ac.uk/projects/fastqc/ (accessed on 8 January 2025)) and trimmed to optimize their quality with Trimmomatic v0.39 [31]. Clean reads were uploaded to the PLACNETw plasmid reconstruction tool [32] to assemble and elucidate genome components. All the assemblies were evaluated using QUAST 5.2.0 (Table S2). In silico predictions of *E. coli* muti-locus sequence type (MLST) profiles were conducted utilizing the Achtman 7 gene scheme via EnteroBase (http://enterobase.warwick.ac.uk (accessed on 17 September 2024)). AMR and the virulence gene content were estimated with ResFinder v4.6 and VirulenceFinder v2.0, or Kleborate v2.3.2, applying thresholds of 90% identity and 60% minimum length, respectively [33,34,35]. According to the phylogenomic study, two ESBL/CP-Ec/Kp core-genus phylogenies (cgMLSTs) were constructed.

### 2.3. Comparative Phylogenomics of ESBL/CP-Ec/Kp Clinical Isolates with Those Previously Obtained from the Storks Foraging in Landfills

Forty-four genomes retrieved from ESBL/CP-Ec/Kp clinical isolates (35 Ec-ST10/ST58/ST131 and 9 Kp-ST307) were mapped to the previously analyzed ESBL/CP-Ec/Kp storks’ genomes corresponding to similar STs (14 Ec storks’ genomes related to ST10/ST58/ST131 and 7 Kp storks’ genomes related to ST307), creating two additional phylogenies. The resulting trees were visualized using iTol v5.5.1 (http://itol.embl.de/itol.cgi (accessed on 10 January 2025)).

### 2.4. Nucleotide Sequence Accession Numbers

Raw sequence reads reported in this paper were deposited under NCBI BioProject PRJNA1274154.

## 3. Results

### 3.1. Distribution of ESBL and CP Types Among Ec/Kp Clinical Isolates

The following combinations of ESBL and/or CP genes were identified: (i) for Ec, *bla*_CTX-M-15_ (n = 32, 57.1%), *bla*_CTX-M-27_ (n = 10, 17.9%), *bla*_CTX-M-14_ (n = 7, 12.5%), *bla*_CTX-M-9_ (n = 2, 3.6%), *bla*_CTX-M-1_ (n = 1, 1.8%), *bla*_CTX-M-55_ (n = 1, 1.8%), *bla*_CTX-M-88_ (n = 1, 1.8%), *bla*_CTX-M-15_+*bla*_CTX-M-27_ (n = 1, 1.8%), and *bla*_NDM-5_ (n = 1, 1.8%); (ii) for Kp, *bla*_CTX-M-15_ (n = 31), *bla*_CTX-M-1_ (n = 1), *bla*_CTX-M-9_ (n = 1), *bla*_CTX-M-14_ (n = 2), *bla*_OXA-48_+*bla*_CTX-M-15_ (n = 13), *bla*_KPC-3-like_ (n = 13), and *bla*_OXA-48_ (n = 4) (Table 1). In the case of Kp-*bla*_KPC-3-like_, we only considered it in the descriptive analysis, as the WGS data were already reported in our previous study [30].

### 3.2. Molecular Epidemiology of E. coli Clinical Isolates

A comprehensive molecular characterization of 44 clinical ESBL/CP-Ec isolates (n = 43 ESBL-Ec, n = 1 CP-Ec) selected from the total 56 ESBL/CP-Ec isolates recovered in this study revealed a predominant presence of ESBL dominated by *bla*_CTX-M_ genes. The subset of Ec isolates selected for WGS comprised a collection that exhibits a diverse range of *bla*_ESBL_ genes, as previously described. The selection criteria were based on MLVA profiles to ensure genetic heterogeneity among the strains (see Section 2.1). Class one integrons (*intI1*) were detected in 56.8% (n = 25/43) of ESBL-Ec isolates, often associated with conserved resistance cassettes such as *dfrA17-aadA5*, *dfrA14*, *dfrA25*, and *dfrA1-aadA1*. Sulfonamide resistance genes *sul2* and *sul1* were found in 27.3% (n = 12) and 34.8% (n = 15) of isolates, respectively (Table 2). Outside the integron, we found a diversity of AMR genes. Firstly, those codifying aminoglycoside- and fluoroquinolone-modifying enzymes were among the most common, including *aac(6′)-Ib-cr* in 52.3% (n = 23), *aac(3)-IIa* in 40.9% (n = 18), and *aac(3)-IId* in 9.1% (n = 4). Secondly, the phosphotransferase genes *aph(3″)-Ib* and *aph(6)-Id* were each found in 34.1% (n = 15) and 27.3% (n = 12) of isolates. Thirdly, resistance to macrolides via the *mph*(*A*) gene was present in 29.5% of isolates (n = 13), while chloramphenicol resistance gene *catB3* was observed in 52.3% (n = 23). Finally, tetracycline resistance genes were also prevalent, with *tet(A)* detected in 54.5% (n = 24) and *tet(B)* in 6.8% of isolates (n = 3). In addition, plasmid-mediated quinolone resistance determinants *qnrS1* (25.0%, n = 11) and *qnrB19* (4.5%, n = 2) were also present (Table 2). Plasmid replicon typing revealed the widespread presence of multi-replicon IncF-type plasmids, with IncFIA-IncFIB-IncFII combinations identified in 54.5% (n = 24) of isolates. Additional replicon types included IncFIA-IncFIB (29.5%, n = 13), Col156 (34.1%, n = 15), Col(BS512) (22.7%, n = 10), IncN (31.8%, n = 14), IncI1-I (13.6%, n = 6), and IncX1 or IncX4 (13.6%, n = 6) (Table 2). The co-occurrence of these plasmid types, many of which are known to carry both resistance and virulence determinants, highlights their key role in the horizontal dissemination of multidrug resistance. Virulence profiling demonstrated a high prevalence of traits characteristic of both uropathogenic Ec (UPEC) and extraintestinal pathogenic Ec (ExPEC). Iron acquisition systems were nearly universal, with siderophore-related genes (*entA-F*, *fepA-G*, *fes*, *irp1*, *irp2*, *iucA-D*, *iutA*) found in over 90% of isolates. The hemo transport operon *chuA-Y* was detected in 86.4% (n = 38). Adhesins were also highly represented, with the complete *fim* operon (*fimA-I*) found in 88.6% (n = 39) and *pap* genes (*papC-K*) in 72.7% (n = 32). The *ecp* operon (*ykgK/ecpR*), involved in adherence and biofilm formation, was well conserved across the dataset. Toxin-encoding genes such as *sat* (36.4%, n = 16), *hlyA-D* (29.5%, n = 13), *cnf1* (20.5%, n = 9), and *vat* (13.6%, n = 6) were variably distributed. Genes related to capsule formation and serum resistance, including *ompA*, *kpsD*, and *kpsM*, were found in more than 90% of isolates. Based on the presence of ≥2 ExPEC-defining markers (i.e., *fyuA*, *iutA*, *ompA*, *papC*, *kpsMII*), 84.1% (n = 37) of the isolates could be classified as ExPEC and/or UPEC (Table 2).

### 3.3. Phylogenomic Analysis and Plasmid Location of ESBL/CP-E. coli Clinical Isolates

**Figure 1:**
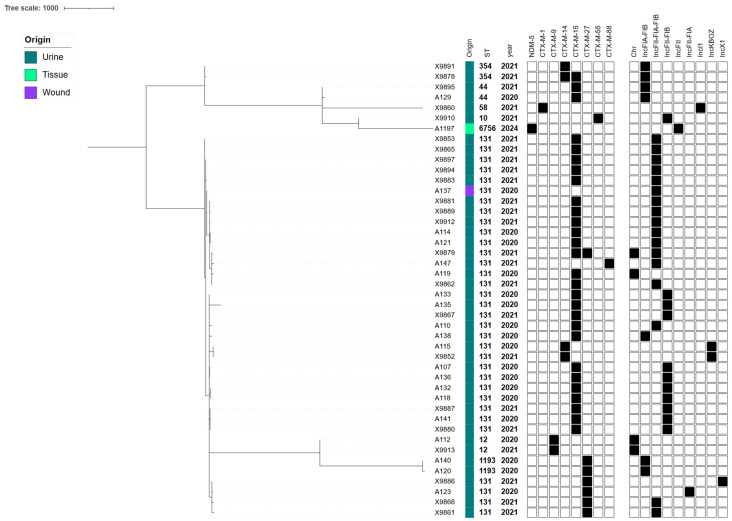
cgMLST-based phylogeny of the 44 ESBL/CP-Ec isolates collected from human infections during 2020 to 2024. The Metadata columns include the origin of the infection shown with colored bars (each color corresponds to a different location), sequence type, and year of isolation. ESBL/CP genes and plasmid contents are indicated with rectangles denoting a gene’s presence (filled shapes) or absence (empty shapes).

The cgMLST analysis of the 44 ESBL/CP-Ec clinical isolates submitted to WGS revealed a phylogenomic-structured population dominated by high-risk clonal lineages. The most frequently identified sequence type (ST) was ST131, accounting for 75.0% (n = 33) of the total isolates (Figure 1). This globally disseminated ExPEC lineage exhibited significant diversity and was further subdivided into three distinct subclades based on cgMLST allelic variation. Within ST131, from 0 to over 200 ADs were observed, indicating both recent clonal expansion events (evidenced by clusters differing by <50 ADs) and broader intra-lineage genetic diversification (Figure 1, Table S3). Other STs were less frequently represented but demonstrated significant phylogenetic contributions. ST1193, another emerging pandemic ExPEC clone, was identified in 4.5% (n = 2) of the isolates and clustered tightly in the cgMLST phylogeny (<100 Ads), suggesting recent expansion and local dissemination (Figure 1, Table S3). ST12, ST44, and ST354 were each represented by two isolates (4.5%), while ST10, ST58, and ST6756 appeared as singletons (2.3% each) (Figure 1). The ST6756 isolate, notable for being the only CP producer in the collection, displayed over 1500 ADs from all other strains, confirming its distinct evolutionary origin (Table S3). The genomic location of ESBL genes revealed that most of these resistance genes were plasmid-borne. Firstly, *bla*_CTX-M-15_, the most prevalent ESBL gene, was consistently located on IncF-type plasmids, particularly those with multi-replicon configurations (IncFIA-IncFIB-IncFII). This plasmid profile was especially dominant in ST131 isolates, with confirmed co-localization of *bla*_ESBL_ in over 90% of *bla*_CTX-M-15_-positive isolates (Figure 1). Maintaining these multi-replicon plasmids within ST131 lineages supports their critical role in both the dissemination and stable inheritance of resistance traits. Secondly, *bla*_CTX-M-27_ was frequently located in association with IncFIA-IncFIB and Col156 replicons (Figure 1). Thirdly, *bla*_CTX-M-14_, present in phylogenetically unrelated STs, was likewise plasmid-encoded but demonstrated greater diversity in replicon context, including associations with IncFIA-FIB or IncKBOZ plasmids, highlighting its capacity for horizontal transfer across diverse genetic backgrounds (Table 2). The single *bla*_NDM-5_ gene was found in an ST6756 isolate and mapped to a plasmid carrying IncX1 and IncFII replicons, plasmid types commonly involved in the global dissemination of CP genes. This isolate also carried other multiple resistance markers, confirming the accumulation of multidrug resistance within a mobile plasmid context (Table 2, Figure 1).

### 3.4. Cross-Sectional Genomic Analysis of ESBL/CP-Ec Clinical Isolates vs. ESBL/CP-Ec from Storks Feeding on a Nearby Landfill

**Figure 2:**
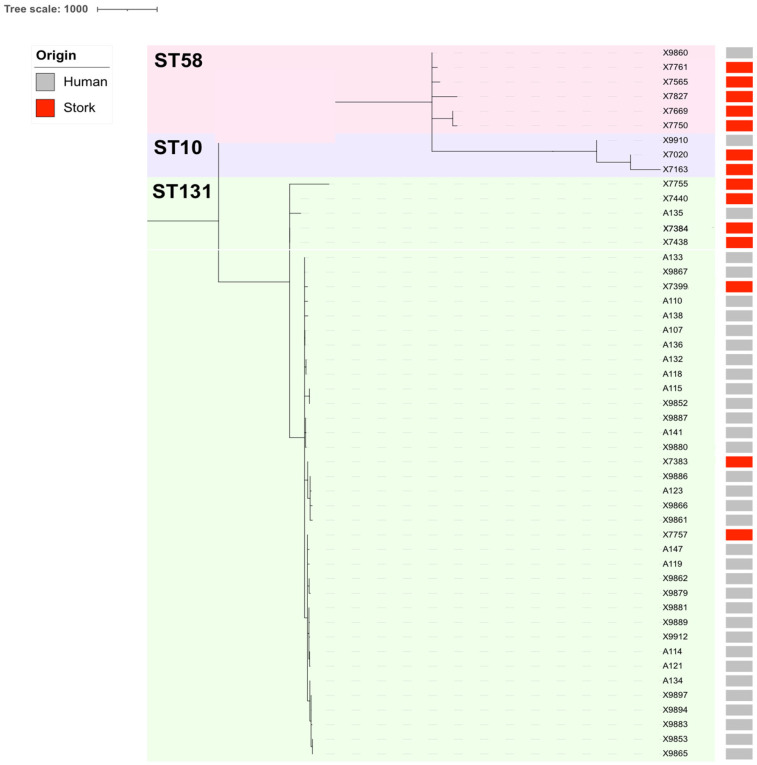
Comparative phylogenomics of the ESBL/CP-Ec isolates linked to ST10, ST58, and ST131 collected from human infections during 2020 to 2024, as well as the ESBL/CP-Ec isolates previously recovered from storks’ feces during 2020 to 2021 [30]. Metadata columns include origin with colored bars (gray: human infections; red: storks’ feces).

Additionally, a comparative cgMLST analysis of the clinical ESBL/CP-Ec isolates linked to ST10, ST58, and ST131 (n = 35; 2020–2024), as well as those previously recovered from white storks’ feces of the same STs (n = 14; 2020–2021), revealed meaningful phylogenetic relationships. Overall, clustering patterns indicated both host-specific lineage divergence and evidence of inter-host or environmental transmission. Among ST10 isolates, moderate clonality (105 to 128 ADs) was observed between clinical and stork strains, suggesting a more distant common ancestor and limited recent exchange (Table S4, Figure 2). Additionally, a subset of isolates belonging to ST58 retrieved from storks’ feces displayed more distant relationships (129–136 ADs) with our X9860 Ec clinical isolate (Table S4, Figure 2). In contrast, ST131 isolates exhibited substantially higher relatedness. For example, the stork isolate X7757 differed by only 22 ADs with the clinical isolate A121; moreover, the isolate X7757 differed 39, 50, and 68 ADs with the clinical isolates A132, A133, and A134, respectively (Table S4). These findings were supported by the cgMLST-based clade structure, which revealed two major ST131 subclades with both clinical and stork-derived isolates intermingled within the same phylogenetic clusters (Figure 2).

### 3.5. Molecular Epidemiology of ESBL/CP-Kp Clinical Isolates

Of the 65 ESBL/CP-Kp isolates analyzed in this study, 18 were newly sequenced as part of this work, while an additional group of 13 Kp/*bla*_KPC-3-like_ isolates had been previously sequenced as described (see Section 2) [30]. The 18 ESBL/CP-Kp clinical isolates sequenced reveal a predominance of high-risk clones and a diverse set of AMR genes, including *bla*_OXA-48_ with *bla*_CTX-M-15_ (Table 3).

The Kp-isolates were distributed across six sequence types (STs), with ST307 being the most common (44.4%, n = 8), followed by ST11 (22.2%, n = 4), ST15 (22.2%, n = 4), and single representatives of ST392 and ST326. The K-locus (KL) and *wzi* typing showed a correlation with STs: ST307 was consistently associated with KL102 and *wzi173*; ST11 and ST15 were linked to KL24 and *wzi24*; and ST392 or ST326 corresponded to KL27 and KL25, respectively (Table 3). Integron analysis revealed the presence of class one integrons (*intI1*) in 38.9% (n = 7) of the isolates, including in their variable region different arrays, such as *dfrA14* and *dfrA15-aadA1-qacEΔ1-sul1,* among others. Non-β-lactam resistance genes were highly prevalent. Aminoglycoside resistance was conferred by *aac(3)-IIa*, *aac(6’)-Ib-cr*, *strA*, and *strB* genes, found in >60% of isolates. Fluoroquinolone resistance was associated with *qnrB1* (30%, n = 6), while the *catB4* (55.6%, n = 10) and *catA1* genes (22.2%, n = 4) conferred chloramphenicol resistance. Sulfonamide resistance genes *sul2* and *sul1* were found in 61.1% and 22.2% of isolates, respectively. The tetracycline resistance gene *tet(A)* was present in 33.3%, and trimethoprim resistance genes *dfrA14* and *dfrA15* were detected in 21.4% and 22.2% of isolates, respectively (Table 3).

Plasmid analysis revealed a diverse and complex plasmidome among the ESBL/CP-Kp isolates. The most frequently detected replicons included IncFIB(K) and ColpVC, present in a high proportion of the isolates, often co-occurring with IncL and IncR plasmids. Several isolates also harbored IncHI1B, IncN, IncFIA, and multi-replicon configurations such as IncHI1B-FIB, indicating significant plasmid modularity. Notably, many isolates exhibited combinations of three to five plasmid types, highlighting the plasmid-mediated plasticity that contributes to the persistence and spread of multidrug resistance (Table 3).

Virulence profiling revealed the presence of the *ybt* (yersiniabactin) siderophore locus in 30% (n = 6) of isolates, specifically *ybt13* (ST307) and *ybt10* (ST11). These were integrated into ICEKp structures such as ICEKp2 and ICEKp4. All of these isolates lacked hypervirulence or hypermucoviscous markers (i.e., *rmpA*, *rmpA2*, *iro*, *iuc*), supporting their classification as classical MDR-Kp (Table 3).

### 3.6. Phylogenomic Analysis and Plasmid Location of ESBL/CP-Kp Isolates with a Cross-Sectional Study

**Figure 3:**
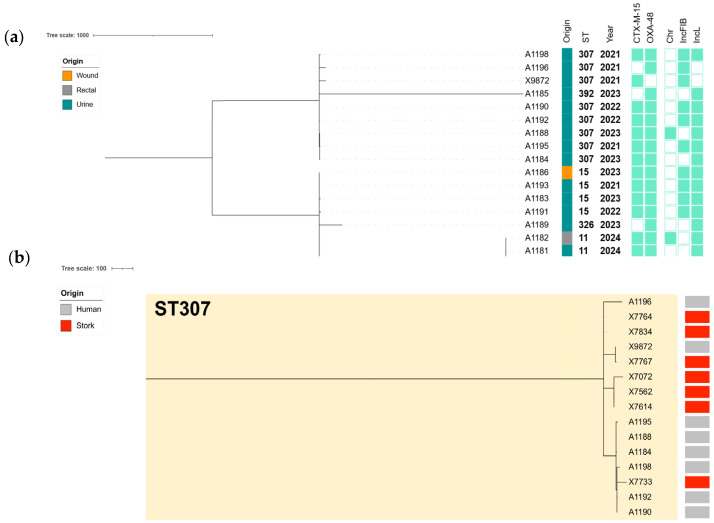
(**a**) cgMLST-based phylogeny of the 18 ESBL/CP-Kp isolates collected from human infections during 2020 to 2024. The Metadata columns include the origin of the infection shown with colored bars (each color corresponds to a different location), sequence type, and year of isolation. ESBL/CP genes and plasmid contents are indicated with rectangles denoting a gene’s presence (filled shapes) or absence (empty shapes). (**b**) Comparative phylogenomics of the ESBL/CP-Kp isolates linked to ST307 collected from human infections during 2020 to 2024, as well as the ESBL/CP-Kp isolates previously recovered from storks’ feces during 2020 to 2021 [30]. The metadata columns include origin with colored bars (gray: human infections; red: storks’ feces).

The cgMLST analysis of the 18 ESBL/CP-Kp clinical isolates revealed a structured population consisting predominantly of high-risk clones, particularly ST307, ST11, and ST15. ST307 was the most prevalent sequence type, accounting for 44.4% (n = 8 of the isolates), followed by ST11 (22.2%, n = 4) and ST15 (22.2%, n = 4) (Figure 3a). Single representatives were also identified for ST392 and ST326 (Table S5), delineating multiple clades with ST-specific clustering and supporting intra-ST diversification over time (Figure 3).

This analysis showed clear evidence of clonal spread and microevolution. Within ST307, several isolates were highly related, with pairwise allelic differences of ≤10. Specifically, A1190 and A1192 differed by only three ADs, or A1190 and A1188 differed by just four ADs. A broader group of closely related ST307 isolates, including X9872, A1190, and A1196, showed pairwise distances ranging from 9 to 71 ADs, suggesting recent common ancestry and possible clonal dissemination. In contrast, ST11 and ST15 isolates were more distantly related to each other and to ST307, with inter-ST distances exceeding 1700 ADs, confirming distinct clonal lineages (Figure 3a, Table S5).

### 3.7. Cross-Sectional Genomic Analysis of ESBL/CP-Kp Clinical Isolates vs. ESBL/CP-Kp from Storks Feeding on a Nearby Landfill

Additionally, a cross-sectional cgMLST analysis of the high-risk clone ST307-Kp clinical isolates (n = 8) and those previously recovered from white stork feces (n = 7) during 2020–2021 [30] revealed strong genetic relatedness. The phylogenetic tree (Figure 3b) highlighted two principal subclades composed of both human- and stork-derived isolates, suggesting clonal continuity across host sources and settings. Our analysis confirmed the presence of several near-identical strain pairs with ≤20 ADs, a threshold commonly interpreted as indicative of recent transmission (Table S6). Notably, human isolate X9872 and stork isolate X7767 showed five ADs (Figure 3b, Table S6). Additional closely related human–stork pairs included A1195-X7072 (14 ADs), A1196-X7733 (16 ADs), and A1190-X7767 (18 ADs), indicating potential cross-host or environmental circulation of highly similar clones (Figure 3b, Table S6). Plasmid mapping showed that ESBL and CP genes, particularly *bla*_CTX-M-15_ and *bla*_OXA-48_, were consistently plasmid-encoded, with IncL plasmids being common vectors in both human and wildlife settings, due to their identification in 85% of the Kp isolates. Among ST307 isolates, a highly conserved plasmid profile was observed, characterized by the recurrent detection of IncFIB(K) mainly associated with the dissemination of *bla*_CTX-M-15_ [30].

## 4. Discussion

This study provides a genomic and epidemiological snapshot of ESBL/CP-Ec/Kp isolates collected in a tertiary Spanish hospital between 2020 and 2024. Our findings align with trends reported in national and European surveillance programs, reinforcing the interconnectedness of clinical, environmental, and wildlife reservoirs in the dissemination of AMR. In the early study period (2020–2021), the predominance of ESBL-Ec/Kp isolates, especially those harboring *bla*_CTX-M-15_ and *bla*_CTX-M-14_, mirrors the epidemiology described in Spain’s GEIH-BLEE multicenter project during 2006 (44 hospitals documented high rates of ESBL-Ec/Kp, with *bla*_CTX-M-15_ as the leading determinant in the same line as in our cohort) [36]. Similarly, an increased prevalence of CPE was reported, predominantly marked by Kp-*bla*_OXA-48_. This was reflected in the national CARB-ES-19 study, which reported an increased prevalence and geographical spread of OXA-48-producing ST307 and ST11 clones in Spanish hospitals [6,7]. Notably, we identified a single Ec-ST6756 isolate carrying *bla*_NDM-5_, a rare finding in Spain that likely represents sporadic introduction via mobile genetic elements. Our cgMLST-based analysis confirmed the dominance of international high-risk clones, particularly Ec-ST131 (75.0%) and Kp-ST307 (44.4.%), both well-established epidemic lineages associated with *bla*_CTX-M-15_ carriage and multidrug resistance [37,38]. Intra-ST variability revealed clonal expansions (≤10 ADs) among some ST131 and ST307 isolates, suggesting localized transmissibility or selection under AMR pressure. In contrast, the appearance of genetically distant CP producers with divergent plasmid profiles (i.e., IncX1 or IncFII plasmid carrying *bla*_NDM-5_) underscores the role of horizontal gene transfer in the emergence of resistance. In our study, class one integrons were detected in over half of the Ec and Kp isolates, typically associated with AMR genes cassettes such as *dfrA17-aadA5*, *dfrA14*, and *aadA1*, consistent with integron architectures described in Spanish clinical isolates [39]. Plasmid replicon typing further confirmed the prominence of IncFIB(K) and IncL plasmids, particularly among *bla*_CTX-M-15/_*bla*_KPC-3_ and *bla*_OXA-48_ carriers, supporting previous findings from Spain and Portugal [40]. The co-existence of resistance and virulence genes on multi-replicon plasmids (i.e., IncFIA-IncFIB-IncFII) facilitates the persistence and transmission of these traits across compartments. Beyond clinical settings, several studies have highlighted the circulation of ESBL/CP-Ec in wildlife and environmental reservoirs in Spain. For example, our group has previously reported OXA-48- and CTX-M-15-producing Ec and Kp in white stork feces foraging at landfills in central Spain, including ST131 and ST307 isolates with ≤20 ADs to our clinical strains, suggesting inter-reservoir dissemination [30]. Similar findings have been reported in Dutch wild birds where ESBL-Ec linked to ST10, ST58, and ST69 detected in wild bird feces often shared an AMR plasmid profile with clinical isolates [41]. Livestock environments, particularly poultry and pig farms, have also been recognized as sources of high-risk clones and resistance genes, including *bla*_CTX-M-1_ and *bla*_CTX-M-14_ [42,43,44]. Furthermore, wastewater-based surveillance in Spain has repeatedly identified ESBL/CPE, including Ec-ST131 and Kp-ST15/ST307, highlighting wastewater [45] or the clinical backdrop [46] as a key interface for environmental dissemination.

Our cgMLST analysis of 44 ESBL/CP-producing Ec isolates revealed a structured population dominated by globally disseminated ExPEC clones, in particular, ST131 (75%), which showed considerable allelic diversity, forming three subclades with intra-clade distances ranging from 0 to >200 ADs. This high prevalence and clonal expansion of ST131 mirrors previous multicentric studies in Spain [6,7] and across Europe [12], where ST131 has been repeatedly linked to healthcare-associated infections and MDR patterns. Similar subclade clustering within ST131 has been reported in both clinical and long-term care facilities in Spain [6,7,36], reinforcing its evolutionary success under antibiotic pressure. The second most prevalent clone in our study, ST1193 (4.5%), is a fluoroquinolone-resistant ExPEC lineage that has recently been emerging in both hospital and community settings. The tight clustering of our ST1193 isolates (<100 ADs) suggests recent clonal expansion, consistent with findings in northern Spain [47], where ST1193 was identified as an emerging cause of UTIs. Plasmid analysis confirmed that most ESBL and CP genes were plasmid-borne. IncF-type multi-replicon plasmids (IncFIA-FIB-FII) were predominant among *bla*_CTX-M-15_-positive ST131 isolates, in line with previous work in Spanish hospitals [48] and wastewater environments [49].

To explore the potential ecological and evolutionary interface between human and environmental reservoirs, we performed a cgMLST-based comparison of 35 clinical ESBL-Ec isolates (ST10, ST58, ST131) with 14 ESBL-Ec isolates previously recovered from white stork’s fecal samples with landfill foraging collected at the municipal landfill near to the city in which the hospital is located in south–central Spain [30]. Overall, the phylogenetic clustering revealed notable inter-host genomic relatedness, with several clinical and stork isolates belonging to the same STs and separated by <25 ADs, suggesting recent common ancestry or inter-reservoir exchange. Among ST10 isolates, allelic differences between clinical and stork strains ranged from 105 to 128 ADs, indicating evolutionary relatedness but suggesting a more distant divergence, likely driven by separate adaptations in their respective environments. These results are in line with prior findings from wild boars and gulls in the Antarctic [50] and Italy [51], respectively, where ST10 and ST58 were detected in both wildlife and human-associated samples, often differing by more than 100 SNPs. In contrast, ST58 isolates from both clinical and stork sources showed higher genomic similarity. For example, our clinical isolate A121 and stork isolate X7757 differed by only 22 ADs. Other comparisons within ST58 remained below 70 ADs, a threshold compatible with recent clonal spread. ST58 has also been identified in Spanish pig farms [52] and urban water niches in Portugal [53,54], supporting its circulation across diverse ecosystems. The most substantial evidence of inter-reservoir relatedness was observed in ST131, where several clinical isolates (A132, A133, A134, A114, A121) differed from stork isolate X7757 by only 22–68 ADs. These values fall within thresholds for recent transmission or shared environmental exposure. Moreover, most stork and clinical isolates shared conserved plasmid backbones, including multi-replicon IncF (IncFIA-FIB-FII) plasmids associated with *bla*_CTX-M-15_. This plasmid architecture, detected across both reservoirs, further supports the plasmid-mediated dissemination of AMR genes across ecological compartments [55].

The pattern in Kp infections was slightly different. The genomic characterization of the 18 ESBL/CP-Kp isolates (*bla*_KPC-3-like_ isolates were excluded because they were already analyzed) revealed a structured population dominated by high-risk clonal lineages and enriched with MDR determinants. The *bla*_CP_ genes were also highly prevalent, with *bla*_OXA-48_ detected in 17 out of 18 Kp isolates. This aligns with the CARB-ES-19 multicenter study, which identified *bla*_OXA-48_ as the dominant CP gene among Kp in Spain, frequently linked to the ST307 and ST15 lineages [6]. The absence of *bla*_NDM_ and *bla*_VIM_ suggests a geographically defined resistance pattern, dominated by OXA-48-producing clones, as well as KPC-3-producing isolates, as found in our previous study [30]. The isolates were distributed across six sequence types, with ST307, ST11, and ST15 being the most common. These clones are part of globally recognized high-risk lineages and have been repeatedly associated with the dissemination of OXA-48 across Europe [56]. In Spain, ST307 has shown a rapid increase in prevalence over the past decade and is now considered one of the most successful MDR-Kp lineages in hospital settings [6,7,8,10].

Virulence analysis confirmed the presence of the yersiniabactin (*ybt*) siderophore locus in 30% of isolates, notably *ybt13* (ST307) and *ybt10* (ST11), associated with ICEKp2 and ICEKp4 integrative elements. This pattern has previously been reported in MDR-Kp isolates lacking hypervirulence markers, supporting their classification as classical MDR lineages rather than hypervirulent variants [57].

Plasmid mapping revealed that both *bla*_CTX-M-15_ and *bla*_OXA-48_ were consistently plasmid-encoded, primarily within IncL, IncFIB(K), and ColpVC replicon backgrounds. The association between *bla*_OXA-48_ and IncL plasmids has been widely recognized across Europe [58,59], and their presence in both ST307 and ST11 in this study further confirms their role in CP dissemination [59]. Additionally, IncHI1B–IncFIB plasmids were detected in high-risk *Klebsiella pneumoniae* clones such as ST15 and ST11. These hybrid plasmids often co-harbor both AMR and virulence genes and have been reported in clinical hypervirulent ST15 isolates as well as in epidemic ST11 CR-hvKp outbreaks (i.e., *rmpA*, *iucABCD*–*iutA*, *bla*_CTX-M_, and *bla*_NDM_) [60]. In accordance with this, for *bla*_KPC-3_, we also evidenced the same high association between the *bla*_CP_ gene and plasmid type with IncF (K1:A-:B-) [30], suggesting the stable association of these plasmids across genetically similar clones and their role in maintaining MDR within this high-risk lineage.

Our cgMLST confirmed the clonal expansion within ST307, with several isolates differing by ≤10 alleles (i.e., X9872 vs. X7767: 5 ADs), suggesting recent local transmission. In contrast, ST11 and ST15 isolates exhibited greater allelic divergence (>1700 ADs), indicating polyclonal introduction events. This structure mirrors Spanish hospital outbreaks involving ST307 clones harboring *bla*_OXA-48_ documented by Cañada-García et al. (2022) and Gracia-Ahufinger et al. (2023) [6,7]. As previously stated in our study [30] for the 13 *bla*_KPC_-positive clinical isolates of the same hospital, it was revealed that 12 of them belonged to a highly conserved ST512/*bla*_KPC-3_ clone, which was also detected in 1 stork-derived isolate (5–19 ADs). Additionally, a cross-sectional phylogenomic cgMLST of our clinical Kp-ST307 isolates with those previously recovered from white storks [30] revealed remarkable genetic similarity, with several human–avian isolate pairs differing by ≤20 alleles (i.e., X9872 vs. X7767: 5 ADs; A1195 vs. X7072: 14 ADs; A1196 vs. X7733: 16 ADs; or A1190 vs. X7767: 18 ADs). These findings support the hypothesis of clonal dissemination of ST307 between human and environmental reservoirs, likely facilitated by shared plasmid structures and anthropogenic interfaces such as landfills and urban wastewater.

## 5. Conclusions

The expanding threat of AMR requires a comprehensive understanding of its drivers, reservoirs, and transmission pathways across human, animal, and environmental domains. This study reinforces the potential need to integrate high-resolution genomic surveillance into clinical and public health frameworks, not only to detect emerging resistance but also to anticipate its spread across ecosystems. The close phylogenetic and plasmid-related links observed between human pathogens and strains circulating in wildlife highlight the blurred boundaries between clinical and environmental reservoirs. These findings underscore the critical importance of adopting a One Health approach that combines molecular epidemiology with cross-sectoral collaboration. Only through coordinated action can we effectively monitor, contain, and ultimately reduce the burden of AMR at the local, national, and global levels.

## Figures and Tables

**Table 1:** Epidemiological data of the 121 ESBL/CP-Ec/Kp isolates retrieved in this study and those selected for WGS.

ID	ESBL/CP/ Detected	Sample Procedence	Origin of Isolation	WGS	N° of Isolates
ESBL-Ec	CTX-M-1	Community	Urine	Yes	1
	CTX-M-9	Community	Urine	Yes	2
	CTX-M-14	Community	Urine	Yes	4
		Nosocomial	Urine	-	2
			Blood culture	-	1
	CTX-M-15	Community	Urine	Yes	26
				-	4
			Wound	Yes	1
		Nosocomial	Urine	-	1
	CTX-M-15+CTX-M-27	Nosocomial	Urine	Yes	1
	CTX-M-27	Community	Urine	Yes	5
				-	1
		Nosocomial	Urine	Yes	1
				-	2
			Wound	-	1
	CTX-M-55	Community	Urine	Yes	1
	CTX-M-88	Community	Urine	Yes	1
CP-Ec	NDM-5	Nosocomial	Tissue (biopsy)	Yes	1
ESBL-Kp	CTX-M-1	Community	Urine	-	1
	CTX-M-14	Community	Urine	-	1
		Nosocomial	Urine	-	1
	CTX-M-9	Community	Urine	-	1
	CTX-M-15	Community	Urine	-	12
	CTX-M-15	Nosocomial	Urine	Yes	1
	CTX-M-15	Nosocomial	Urine	-	15
	CTX-M-15	Nosocomial	Blood culture	-	2
	CTX-M-15	Nosocomial	Wound	-	1
CP-Kp	KPC-3	Community	Abscess	Yes *	1
			Urine		3
		Nosocomial	Blood culture	Yes *	1
			Urine		6
			Wound		1
	KPC-39	Community	Urine	Yes *	1
	OXA-48	Community	Urine	Yes	1
		Nosocomial	Urine	Yes	3
ESBL+CP-Kp	CTX-M-15+OXA-48	Community	Urine	Yes	7
	CTX-M-15+OXA-48	Nosocomial	Rectal	Yes	1
			Urine	Yes	4
			Wound	Yes	1

(*) These Kp/*bla*_KPC-3-like_ isolates were already sequenced in a previously published study [30].

**Table 2:** Genomic features of the 44 ESBL/CP-*E. coli* strains whole-genome sequenced included in this study.

Strain	ST/PG	ESBL/CP/Other Beta- Lactamases	Class 1 Integron		Resistance Genes (Outside the Integron)	Plasmid Content	Virulence Profile
			intI1/ 3′-CS	Variable Region			
A107	131/B2	CTX-M-15	−/−	−	*tet(A)*	IncFIB-FII, IncI1, Col(BS512)	*aslA*, ***chuA*** **^a^**, *chuSTUVWXY*, *csgBDFG*, *entABCDEFS*, *fdeC*, *fepABCDG*, *fes*, *fimACDEFGHI*, ***fyuA*** **^a^**, *gspLM*, *irp12*, *iucABCD*, ***iutA*** **^b^**, *kpsD*, ***kpsM*** **^b^**, *ompA*, ***papC*** **^b^**, *papDFGHIJKX*, *sat*, *yagVWXYZ/ecpEDCBA*, *ybtAEPQSTUX*, *ykgK/ecpR*
A110	131/B2	CTX-M-15, OXA-1, TEM-1B	+/+	*dfrA17*	*aac(3)-IId*, *aac(6′)-Ib-cr*, *aph(3″)-Ib*, *aph(6)-Id*, *catB3*, *mph*(A), *sul2*, *tet*(A)	IncFIA-IncFII, Col156	*aslA*, ***chuA*** **^a^**, *chuSTUVWXY*, *csgBDFG*, *entABCDEFS*, *fdeC*, *fepABCDG*, *fes*, *fimACDEFGHI*, ***fyuA*** **^a^**, *gspLM*, *irp12*, *iucABCD*, ***iutA*** **^b^**, *kpsD*, ***kpsM*** **^b^**, *ompA*, ***papC*** **^b^**, *papDFGHIJKX sat*, *yagVWXYZ/ecpEDCBA*, *ybtAEPQSTUX*, *ykgK/ecpR*
A114	131/B2	CTX-M-15, OXA-1, TEM-1B	+/+	*dfrA25*	*aac(3)-IIa*, *aac(6′)-Ib-cr*, *catB3*, *qnrB2*, *tet*(A)	IncFIA-FIB, IncN, Col156	*aslA*, ***chuA*** **^a^**, *chuSTUVWXY*, *cnf1*, *csgBDFG*, *entABCDEFS*, *fdeC*, *fepABCDG*, *fes*, *fimACDEFGHI*, ***fyuA*** **^a^**, *gspLM*, *hlyABCD*, *irp12*, *iucABC*, *kpsD*, ***kpsM*** **^b^**, *ompA*, ***papC*** **^b^**, *papDFGHIJKX*, *sat*, *senB*, *yagVWXYZ/ecpEDCBA*, *ybtAEPQSTUX*, *ykgK/ecpR*
A115	131/B2	CTX-M-14, OXA-1, TEM-1C	+/−	*dfrA17-aadA5*	*aac(6′)-Ib-cr*, *aph(3″)-Ib*, *aph(6)-Id*, *catB3*, *sul2*, *tet*(A)	IncB/O/K/Z, IncFIA	*aslA*, ***chuA*** **^a^**, *chuSTUVWXY*, *csgBDFG*, *entABCDEFS*, *fdeC*, *fepABCDG*, *fes*, *fimACDEFGHI*, ***fyuA*** **^a^**, *irp12*, *iucABCD*, ***iutA* ^b^**, *kpsD*, *ompA*, *papB*, ***papC*** **^b^**, *papDFGHIJKX*, *sat*, *yagVWXYZ/ecpEDCBA*, *ybtAEPQSTUX*, *ykgK/ecpR*
A118	131/B2	CTX-M-15, OXA-1	−/−	−	*aac(3)-IIa*, *aac(6′)-Ib-cr*, *catB3*	IncFIB-FII, Col(BS512), Col156	*aslA*, ***chuA*** **^a^**, *chuSTUVWXY*, *csgBDFG*, *entABCDEFS*, *fdeC*, *fepABCDG*, *fes*, *fimACDEFGHI*, ***fyuA*** **^a^**, *gspLM*, *irp12*, *iucABCD*, ***iutA*** **^b^**, *kpsD*, ***kpsM*** **^b^**, *ompA*, ***papC*** **^b^**, *papDFGHIJKX*, *sat*, *yagVWXYZ/ecpEDCBA*, *ybtAEPQSTUX*, *ykgK/ecpR*
A119	131/B2	CTX-M-15, OXA-1	−/−	−	*aac(3)-IIa*, *aac(6′)-Ib-cr*, *catB3*	−	*aslA*, ***chuA*** **^a^**, *chuSTUVWXY*, *cnf1*, *csgBDFG*, *entABCDEFS*, *fdeC*, *fepABCDG*, *fes*, *fimACDEFGHI*, ***fyuA*** **^a^**, *gspLM*, *hlyABCD*, *irp12*, *iucABCD*, ***iutA*** **^b^**, *kpsD*, ***kpsM*** **^b^**, *ompA*, ***papC*** **^b^**, *papDFGHIJKX*, *sat*, *yagVWXYZ/ecpEDCBA*, *ybtAEPQSTUX*, *ykgK/ecpR*
X9912	131/B2	CTX-M-15, OXA-1	+/+	*dfrA17-aadA5*	*aac(3)-IIa*, *aac(6′)-Ib-cr*, *catB3*, *mph(A)*, *tet(A)*	IncFIA-FIB, Col156	*aslA*, ***chuA*** **^a^**, *chuSTUVWXY*, *cnf1*, *csgBDFG*, *entABCDEFS*, *fdeC*, *fepABCDG*, *fes*, *fimACDEFGHI*, ***fyuA*** **^a^**, *hlyABCD*, *irp12*, ***iutA*** **^b^**, *ompA*, *papB*, ***papC*** **^b^**, *papDFGHIJKX*, *senB*, *yagVWXYZ/ecpEDCBA*, *ybtAEPQSTUX*, *ykgK/ecpR*
X9897	131/B2	CTX-M-15, OXA-1	−/−	−	*aac(3)-IIa*, *aac(6′)-Ib-cr*, *catB3*, *tet(A)*	IncFIA-FIB, IncX4	*aslA*, ***chuA*** **^a^**, *chuSTUVWXY*, *csgBDFG*, *entABCDEFS*, *fdeC*, *fepABCDG*, *fes*, *fimACDEFGHI*, ***fyuA*** **^a^**, *irp12*, *ompA*, *papB*, ***papC*** **^b^**, *papDFGHIJKX*, *yagVWXYZ/ecpEDCBA*, *ybtAEPQSTUX*, *ykgK/ecpR*
A121	131/B2	CTX-M-15	+/+, +/−	*dfrA17-aadA5*; *dfrA14*	*mph(A)*, *qnrS1*	IncFIA-IncFIB, IncN, Col156	***chuA*^a^**, *chuSTUVWXY*, *cnf1*, *csgBDFG*, *entABCDEFS*, *fdeC*, *fepABCDG*, *fes*, *fimACDEFGHI*, ***fyuA*** **^a^**, *gspLM*, *hlyABCD*, *irp12*, *iucAB*, *iucC*, *kpsD*, ***kpsM*** **^b^**, *ompA*, ***papC*** **^b^**, *papDFGHIJKX*, *sat*, *senB*, *yagVWXYZ/ecpEDCBA*, *ybtAEPQSTUX*, *ykgK/ecpR*
A123	131/B2	CTX-M-27, TEM-30	+/+	*dfrA17-aadA5*	*aph(3″)-Ib*, *aph(6)-Id*, *mph(A)*, *sul2*, *tet(A)*	IncFIA-FII, IncN, IncX1, Col156	*aslA*, ***chuA*** **^a^**, *chuSTUVWXY*, *csgBDFG*, *entABCDEFS*, *fdeC*, *fepABCDG*, *fes*, *fimACDEFGHI*, ***fyuA*** **^a^**, *gspCDEFGHIJKLM*, *irp12*, *iucABCD*, ***iutA*** **^b^**, *kpsD*, ***kpsM*** **^b^**, *ompA*, *papBIX*, *sat*, *yagVWXYZ/ecpEDCBAybtAEPQSTUX*, *ykgK/ecpR*
A132	131/B2	CTX-M-15, OXA-1	−/−	−	*aac(3)-IIa*, *aac(6′)-Ib-cr*, *catB3*	IncFIB-FII, Col(BS512), Col156	*aslA*, ***chuA*** **^a^**, *chuSTUVWXY*, *csgBDFG*, *entABCDEFS*, *fdeC*, *fepABCDG*, *fes*, *fimACDEFGHI*, ***fyuA*** **^a^**, *gspLM*, *irp12*, *iucABCD*, ***iutA*** **^b^**, *kpsD*, ***kpsM*** **^b^**, *ompA*, ***papC*** **^b^**, *papDFGHIJKX*, *sat*, *yagVWXYZ/ecpEDCBA*, *ybtAEPQSTUX*, *ykgK/ecpR*
A133	131/B2	CTX-M-15	−/−	−	*tet(A)*	IncFIB-FII, IncI1, Col(BS512)	*aslA*, ***chuA*** **^a^**, *chuSTUVWXY*, *csgBDFG*, *entABCDEFS*, *fdeC*, *fepABCDG*, *fes*, *fimACDEFGHI*, ***fyuA*** **^a^**, *gspLM*, *irp12*, *iucABCD*, ***iutA*** **^b^**, *kpsD*, ***kpsM*** **^b^**, *ompA*, ***papC*** **^b^**, *papDFGHIJKX*, *sat*, *senB*, *yagVWXYZ/ecpEDCBA*, *ybtAEPQSTUX*, *ykgK/ecpR*
A134	131/B2	CTX-M-15, OXA-1	+/+	*dfrA17-aadA5*	*aac(3)-IIa*, *aac(6′)-Ib-cr*, *catB3*, *mph(A)*, *tet(A)*	IncFIA-FIB	*aslA*, ***chuA*** **^a^**, *chuSTUVWXY*, *cnf1*, *csgBDFG*, *entABCDEFS*, *fdeC*, *fepABCDG*, *fes*, *fimACDEFGHI*, ***fyuA*** **^a^**, *gspLM*, *hlyABCD*, *irp12*, *iucABCD*, ***iutA*** **^b^**, *kpsD*, ***kpsM*** **^b^**, *ompA*, ***papC*** **^b^**, *papDFGHIJKX*, *sat*, *yagVWXYZ/ecpEDCBA*, *ybtAEPQSTUX*, *ykgK/ecpR*
A135	131/B2	CTX-M-15, TEM-1B	+/−	*dfrA12-orf-aadA2*	*aac(3)-IId*	IncFII, Col156	*aslA*, ***chuA*** **^a^**, *chuSTUVWXY*, *csgBDFG*, *entABCDEFS*, *espL1*, *fdeC*, *fepABCDG*, *fes*, *fimACDEFGHI*, ***fyuA*** **^a^**, *gspLM*, *hlyABCD*, *irp12*, *iucABCD*, ***iutA*** **^b^**, *kpsD*, ***kpsM*** **^b^**, *ompA*, ***papA*** **^b^**, *papBEGHIX*, *sat*, *senB*, *yagVWXYZ/ecpEDCBA*, *ybtAEPQSTUX*, *ykgK/ecpR*
A136	131/B2	CTX-M-15	−/−	−	*tet(A)*	IncFIB-FII, IncI1, Col(BS512)	*aslA*, ***chuA*** **^a^**, *chuSTUVWXY*, *csgBDFG*, *entABCDEFS*, *fdeC*, *fepABCDG*, *fes*, *fimACDEFGHI*, ***fyuA*** **^a^**, *gspLM*, *irp12*, *iucABCD*, ***iutA*** **^b^**, *kpsD*, ***kpsM*** **^b^**, *ompA*, ***papC*** **^b^**, *papDFGHIJKX*, *sat*, *yagVWXYZ/ecpEDCBA*, *ybtAEPQSTUX*, *ykgK/ecpR*
A138	131/B2	CTX-M-15, TEM-1B	−/−	−	*aac(3)-IId*	IncFIA, Col156	*aslA*, ***chuA*** **^a^**, *chuSTUVWXY*, *csgBDFG*, *entABCDEFS*, *fdeC*, *fepABCDG*, *fes*, *fimACDEFGHI*, ***fyuA*** **^a^**, *gspCDEFGHIJKLM*, *irp12*, *iucABCD*, ***iutA*** **^b^**, *kpsD*, ***kpsM*** **^b^**, *ompA*, *papB*, ***papC*** **^b^**, *papDEFGHIJKX*, *sat*, *senB*, *yagVWXYZ/ecpEDCBA*, *ybtAEPQSTUX*, *ykgK/ecpR*
A141	131/B2	CTX-M-15, OXA-1, TEM-1A	−/−	−	*aac(3)-IIa*, *aac(6′)-Ib-cr*, *catB3*	IncFIB-FII, Col(BS512), Col156	*aslA*, ***chuA*** **^a^**, *chuSTUVWXY*, *csgBDFG*, *entABCDEFS*, *fdeC*, *fepABCDG*, *fes*, *fimACDEFGHI*, ***fyuA*** **^a^**, *gspLM*, *irp12*, *iucABCD*, ***iutA*** **^b^**, *kpsD*, ***kpsM*** **^b^**, *ompA*, ***papC*** **^b^**, *papDFGHIJKX*, *sat*, *yagVWXYZ/ecpEDCBA*, *ybtAEPQSTUX*, *ykgK/ecpR*
A147	131/B2	CTX-M-88, OXA-1	−/−	−	*aac(6′)-Ib-cr*, *catB3*	IncFIA-FIB	*aslA*, ***chuA*** **^a^**, *chuSTUVWXY*, *csgBDFG*, *entABCDEFS*, *fdeC*, *fepABCDG*, *fes*, *fimACDEFGHI*, ***fyuA*** **^a^**, *gspLM*, *irp12*, *iucABCD*, ***iutA*** **^b^**, *kpsD*, ***kpsM*** **^b^**, *ompA*, ***papC*** **^b^**, *papDFGHIJKX*, *sat*, *yagVWXYZ/ecpEDCBA*, *ybtAEPQSTUX*, *ykgK/ecpR*
X9852	131/B2	CTX-M-14, OXA-1, TEM-1C	+/−	*dfrA17-aadA5*	*aac(6′)-Ib-cr*, *aph(3″)-Ib*, *aph(6)-Id*, *catB3*, *sul2*, *tet(A)*	IncB/O/K/Z, IncFIA	*aslA*, ***chuA*** **^a^**, *chuSTUVWXY*, *csgBDFG*, *entABCDEFS*, *fdeC*, *fepABCDG*, *fes*, *fimACDEFGHI*, ***fyuA*** **^a^**, *irp12*, *iucABCD*, ***iutA*** **^b^**, *kpsD*, *ompA*, *papB*, ***papC*** **^b^**, *papDEFGHIJKX*, *sat*, *yagVWXYZ/ecpEDCBA*, *ybtAEPQSTUX*, *ykgK/ecpR*
X9853	131/B2	CTX-M-15, OXA-1	+/−	*dfrA14*	*aac(3)-IIa*, *aac(6′)-Ib-cr*, *catB3*, *qnrS1*, *tet(A)*	IncFIA-FIB, IncN	*aslA*, ***chuA*** **^a^**, *chuSTUVWXY*, *csgBDFG*, *entABCDEFS*, *fdeC*, *fepABCDG*, *fes*, *fimACDEFGHI*, ***fyuA*** **^a^**, *gspLM*, *irp12*, *iucABCD*, ***iutA*** **^b^**, *kpsD*, ***kpsM*** **^b^**, *ompA*, ***papC*** **^b^**, *papDFGHIJKX*, *sat*, *yagVWXYZ/ecpEDCBA*, *ybtAEPQSTUX*, *ykgK/ecpR*
X9861	131/B2	CTX-M-27	+/+	*dfrA17-aadA5*	*aph(3″)-Ib*, *aph(6)-Id*, *mph(A)*, *sul2*, *tet(A)*	IncFIA-FII, IncX4	*aslA*, ***chuA*** **^a^**, *chuSTUVWXY*, *csgBDFG*, *entABCDEFS*, *fdeC*, *fepABCDG*, *fes*, *fimACDEFGHI*, ***fyuA*** **^a^**, *irp12*, *iucABCD*, ***iutA*** **^b^**, *ompA*, *papBIX*, *sat*, *yagVWXYZ/ecpEDCBA*, *ybtAEPQSTUX*, *ykgK/ecpR*
X9862	131/B2	CTX-M-15, OXA-1	+/−	*dfrA14*	*aac(6′)-Ib-cr*, *catB3*, *qnrS1*, *tet(A)*	IncFIA-FIB, IncN, Col156	*aslA*, ***chuA*** **^a^**, *chuSTUVWXY*, *cnf1*, *csgBDFG*, *entABCDEFS*, *fdeC*, *fepABCDG*, *fes*, *fimACDEFGHI*, ***fyuA*** **^a^**, *gspLM*, *hlyABCD*, *irp12*, *iucABCD*, ***iutA*** **^b^**, *kpsD*, ***kpsM*** **^b^**, *ompA*, ***papC*** **^b^**, *papDFGHIJKX*, *sat*, *senB*, *yagVWXYZ/ecpEDCBA*, *ybtAEPQSTUX*, *ykgK/ecpR*
X9865	131/B2	CTX-M-15, OXA-1	+/−	*dfrA14*	*aac(3)-IIa*, *aac(6′)-Ib-cr*, *catB3*, *qnrS1*, *tet(A)*	IncFIA-FIB, IncN	*aslA*, ***chuA*** **^a^**, *chuSTUVWXY*, *csgBDFG*, *entABCDEFS*, *fdeC*, *fepABCDG*, *fes*, *fimACDEFGHI*, ***fyuA*** **^a^**, *gspLM*, *irp12*, *iucABCD*, ***iutA*** **^b^**, *kpsD*, ***kpsM*** **^b^**, *ompA*, ***papC*** **^b^**, *papDFGHIJKX*, *sat*, *yagVWXYZ/ecpEDCBA*, *ybtAEPQSTUX*, *ykgK/ecpR*
X9866	131/B2	CTX-M-27	+/+	*dfrA17-aadA5*	*aph(3″)-Ib*, *aph(6)-Id*, *mph(A)*, *sul2*, *tet(A)*	IncFIA-FII	*aslA*, ***chuA*** **^a^**, *chuSTUVWXY*, *csgBDFG*, *entABCDEFS*, *fdeC*, *fepABCDG*, *fes*, *fimACDEFGHI*, ***fyuA*** **^a^**, *gspCDEFGGIJKLM*, *irp12*, *iucABCD*, ***iutA*** **^b^**, *kpsD*, ***kpsM*** **^b^**, *ompA*, *papBIX*, *sat*, *yagVWXYZ/ecpEDCBA*, *ybtAEPQSTUX*, *ykgK/ecpR*
X9867	131/B2	CTX-M-15	−/−	−	*tet(A)*	IncFIB-FII, Col(BS512), Col156	*aslA*, ***chuA*** **^a^**, *chuSTUVWXY*, *csgBDFG*, *entABCDEFS*, *fdeC*, *fepABCDG*, *fes*, *fimACDEFGHI*, ***fyuA*** **^a^**, *gspLM*, *irp12*, *iucABCD*, ***iutA*** **^b^**, *kpsD*, ***kpsM*** **^b^**, *ompA*, ***papC*** **^b^**, *papDFGHIJKX*, *sat*, *senB*, *yagVWXYZ/ecpEDCBA*, *ybtAEPQSTUX*, *ykgK/ecpR*
X9879	131/B2	CTX-M-15, CTX-M-27, OXA-1	+/−	*dfrA14*	*aac(3)-IIa*, *aac(6′)-Ib-cr*, *aph(3″)-Ib*, *aph(6)-Id*, *catB3*, *sitABCD*, *sul2*, *tet(A)*	IncB/O/K/Z, IncFIA-FIB, IncN	*aslA*, ***chuA*** **^a^**, *chuSTUVWXY*, *cnf1*, *csgDFG*, *entABCDEFS*, *fdeC*, *fepABCDG*, *fes*, *fimACDEFGHI*, ***fyuA*** **^a^**, *gspLM*, *hlyABCD*, *irp12*, *iucABCD*, ***iutA*** **^b^**, *kpsD*, ***kpsM*** **^b^**, *ompA*, ***papC*** **^b^**, *papDFGHIJKX*, *sat*, *yagVWXYZ/ecpEDCBA*, *ybtAEPQSTUX*, *ykgK/ecpR*
X9880	131/B2	CTX-M-15, OXA-1, TEM-1A	−/−	−	*aac(3)-IIa*, *aac(6′)-Ib-cr*, *catB3*	IncFIB-FII, Col(BS512), Col156	*aslA*, ***chuA*** **^a^**, *chuSTUVWXY*, *csgBDFG*, *entABCDEFS*, *fdeC*, *fepABCDG*, *fes*, *fimACDEFGHI*, ***fyuA*** **^a^**, *gspLM*, *irp12*, *iucABCD*, ***iutA*** **^b^**, *kpsD*, ***kpsM*** **^b^**, *ompA*, ***papC*** **^b^**, *papDFGHIJKX*, *sat*, *yagVWXYZ/ecpEDCBA*, *ybtAEPQSTUX*, *ykgK/ecpR*
X9881	131/B2	CTX-M-15, OXA-1, TEM-1B	+/+ +/−	*dfrA17-aadA5* *dfrA14*	*aac(3)-IIa*, *aac(6′)-Ib-cr*, *catB3*, *mph(A)*, *qnrS1*	IncFIA-FIB, IncN, Col156, Col440II	*aslA*, ***chuA*** **^a^**, *chuSTUVWXY*, *cnf1*, *csgBDFG*, *entABCDEFS*, *fdeC*, *fepABCDG*, *fes*, *fimACDEFGHI*, ***fyuA*** **^a^**, *gspLM*, *hlyABCD*, *irp12*, *iucABC*, *kpsD*, ***kpsM*** **^b^**, *ompA*, ***papC*** **^b^**, *papDFGHIJKX*, *sat*, *senB*, *yagVWXYZ/ecpEDCBA*, *ybtAEPQSTUX*, *ykgK/ecpR*
X9883	131/B2	CTX-M-15	−/−	−	*tet(A)*	IncFIA-FIB	*aslA*, ***chuA*** **^a^**, *chuSTUVWXY*, *csgBDFG*, *entABCDEFS*, *fdeC*, *fepABCDG*, *fes*, *fimACDEFGHI*, ***fyuA*** **^a^**, *gspLM*, *irp12*, *iucABCD*, ***iutA*** **^b^**, *kpsD*, ***kpsM*** **^b^**, *ompA*, ***papC*** **^b^**, *papDFGHIJKX*, *sat*, *yagVWXYZ/ecpEDCBA*, *ybtAEPQSTUX*, *ykgK/ecpR*
X9886	131/B2	CTX-M-27, TEM-135	−/−	−	*aph(3″)-Ib*, *aph(6)-Id*, *mph(A)*, *qnrB19*, *sul2*, *tet(A)*	IncFIA-FII, IncI1, IncX1, Col(pHAD28), Col156	*aslA*, ***chuA*** **^a^**, *chuSTUVWXY*, *csgBDFG*, *entABCDEFS*, *fdeC*, *fepABCDG*, *fes*, *fimACDEFGHI*, ***fyuA*** **^a^**, *gspCDEFGHIJKLM*, *irp12*, *iucABCD*, ***iutA*** **^b^**, *kpsD*, ***kpsM*** **^b^**, *ompA*, *papBIX*, *yagVWXYZ/ecpEDCBA*, *ybtAEPQSTUX*, *ykgK/ecpR*
X9887	131/B2	CTX-M-15, OXA-1	+/−	*dfrA14*	*aac(3)-IIa*, *aac(6′)-Ib-cr*, *catB3*, *qnrS1*	IncFIB-FII, IncN, Col(BS512), Col156	*aslA*, ***chuA*** **^a^**, *chuSTUVWXY*, *csgBDFG*, *entABCDEFS*, *fdeC*, *fepABCDG*, *fes*, *fimACDEFGHI*, ***fyuA*** **^a^**, *gspLM*, *irp12*, *iucABCD*, ***iutA*** **^b^**, *kpsD*, ***kpsM*** **^b^**, *ompA*, ***papC*** **^b^**, *papDFGHIJKX*, *sat*, *yagVWXYZ/ecpEDCBA*, *ybtAEPQSTUX*, *ykgK/ecpR*
X9889	131/B2	CTX-M-15, OXA-1, TEM-30	+/+ +/−	*dfrA17-aadA5* *dfrA14*	*aac(3)-IIa*, *aac(6′)-Ib-cr*, *catB3*, *mph(A)*, *qnrS1*	IncFIA-FIB, IncN, Col156	*aslA*, ***chuA*** **^a^**, *chuSTUVWXY*, *cnf1*, *csgBDFG*, *entABCDEFS*, *fdeC*, *fepABCDG*, *fes*, *fimACDEFGHI*, ***fyuA*** **^a^**, *gspLM*, *hlyABCD*, *irp12*, *iucABC*, *kpsD*, ***kpsM*** **^b^**, *ompA*, ***papC*** **^b^**, *papDFGHIJKX*, *sat*, *senB*, *yagVWXYZ/ecpEDCBA*, *ybtAEPQSTUX*, *ykgK/ecpR*
X9894	131/B2	CTX-M-15, OXA-1	−/−	−	*aac(3)-IIa*, *aac(6′)-Ib-cr*, *catB3*, *tet(A)*	IncFIA-FIB, IncX4	*aslA*, ***chuA*** **^a^**, *chuSTUVWXY*, *csgBDFG*, *entABCDEFS*, *fdeC*, *fepABCDG*, *fes*, *fimACDEFGHI*, ***fyuA*** **^a^**, *irp12*, *ompA*, *papB*, ***papC*** **^b^**, *papDFGHIJKX*, *yagVWXYZ/ecpEDCBA*, *ybtAEPQSTUX*, *ykgK/ecpR*
X9895	44/A	CTX-M-15, OXA-1	+/+	*dfrA17-aadA5*	*aac(3)-IIa*, *aac(6′)-Ib-cr*, *catB3*, *mph(A)*, *sitABCD*, *tet(B)*	IncFIA-FIB, IncX4, p0111	*aslA*, *csgBDFG*, *entABCDEFS*, *espL14R1X45Y1*, *fdeC*, *fepABCDG*, *fes*, *fimABCDEFGHI*, *gspCDEFGHIJKLM*, *iucABCD*, ***iutA*** **^b^**, *ompA*, *yagVWXYZ/ecpEDCBA*, *ykgK/ecpR*
A129	44/A	CTX-M-15, OXA-1	−/−	−	*aac(6′)-Ib-cr*, *catB3*, *mph(A)*, *sitABCD*, *tet(B)*	IncFIA-FIB, IncX4	*aslA*, *csgBDFG*, *entABCDEFS*, *espL1*, *espL4*, *espX1*, *espX4*, *espX5*, *espY1*, *fdeC*, *fepABCDG*, *fes*, *fimABCDEFGHI*, ***fyuA*** **^a^**, *gspCDEFGHIJKLM*, *irp12*, *iucABCD*, ***iutA*** **^b^**, *ompA*, *yagVWXYZ/ecpEDCBAybtAEPQSTUX*, *ykgK/ecpR*
X9910	10/A	CTX-M-55, TEM-1B	+/−	*dfrA14*	*aac(3)-IIa*, *aph(3″)-Ib*, *aph(3′)-Ia*, *aph(6)-Id*, *sitABCD*, *sul2*	IncFIB	*aslA*, *csgBDFG*, *entABCDEFS*, *espL14R1X45Y1*, *fdeC*, *fepABCDG*, *fes*, *fimABCDEFGHI*, *gspIJKLM*, *iucABCD*, ***iutA*** **^b^**, *ompA*, *yagVWXYZ/ecpEDCBA*, *ykgK/ecpR*
X9891	354/F	CTX-M-14, TEM-1B	+/+ +/−	*dfrA1-aadA1* *dfrA17*	*aac(3)-IId*, *aph(3″)-Ib*, *aph(6)-Id*, *sitABCD*, *sul2*, *tet(A)*, *tet(B)*	IncFIA-FIB, IncHI2, IncQ1, Col156	*aslA*, *chuSTUVWY*, *csgBDFG*, *entABCDEFS*, *espL14R1X45Y1*, *fdeC*, *fepABCDG*, *fes*, *fimABCDEFGHI*, ***fyuA*** **^a^**, *gspCDEFGHIJKLM*, *ibeA*, *irp12*, *iucABCD*, ***iutA*** **^b^**, *kpsD*, ***kpsM*** **^b^**, *ompA*, *shuAX*, *yagVWXYZ/ecpEDCBA*, *ybtAEPQSTUX*, *ykgK/ecpR*
X9878	354/F	CTX-M-14	+/+	*dfrA1-aadA1*	*aph(3″)-Ib*, *aph(3′)-Ia*, *aph(6)-Id*, *sitABCD*, *sul2*, *sul3*, *tet(A)*	IncFIA-FIB, IncHI2, IncQ1, pKPC-CAV1193	*aslA*, *chuSTUVWY*, *csgBDFG*, *entABCDEFS*, *espL1R1X4Y24*, *fdeC*, *fepABCDG*, *fes*, *fimABCDEFGHI*, ***fyuA*** **^a^**, *gspCDEFGHIJKLM*, *ibeA*, *irp12*, *iucABCD*, ***iutA*** **^b^**, *kpsD*, *ompA*, *shuAX*, *yagVWXYZ/ecpEDCBA*, *ybtAEPQSTUX*, *ykgK/ecpR*
A120	1193/B2	CTX-M-27	−/−	−	*mph(A)*	IncFIA, IncI1, Col(BS512), Col156,	*aslA*, ***chuA*** **^a^**, *chuSTUVWXY*, *csgBDFG*, *entABCDEFS*, *fdeC*, *fepABCDG*, *fes*, *fimABCDEFGHI*, ***fyuA*** **^a^**, *gspCDEFGHIJKLM*, *irp12*, *iucABCD*, ***iutA*** **^b^**, *kpsD*, ***kpsM*** **^b^**, *kpsT*, *ompA*, *papBIX*, *sat*, *senB*, ***vat*** **^a^**, *yagVWXYZ/ecpEDCBA*, *ybtAEPQSTUX*, *ykgK/ecpR*
A140	1193/B2	CTX-M-27	+/−	*dfrA14*	*qnrS1*	IncFIA-FIB, IncI1, IncN, Col(BS512), Col156,	*aslA*, ***chuA*** **^a^**, *chuSTUVWXY*, *csgBDFG*, *entABCDEFS*, *fdeC*, *fepABCDG*, *fes*, *fimABCDEFGHI*, ***fyuA*** **^a^**, *gspCDEFGHIJKLM*, *irp12*, *iucABCD*, ***iutA*** **^b^**, *kpsD*, ***kpsM*** **^b^**, *kpsT*, *ompA*, *papBIX*, *sat*, ***vat*** **^a^**, *yagVWXYZ/ecpEDCBA*, *ybtAEPQSTUX*, *ykgK/ecpR*
A112	12/B2	CTX-M-9, SHV-48, TEM-30	−/−	−	*ant(3″)-Ia*, *qacE*, *qnrS1*, *sul1*	IncN, Col156	*aslA*, ***chuA*** **^a^**, *chuSTUVWXY*, *cnf1*, *csgBDFG*, *entABCDEFS*, *fdeC*, *fepABCDG*, *fes*, *fimABCDEFGHI*, *focH*, *fyuA*, *gspLM*, *hlyABC*, *iroBCDEN*, *irp12*, *iucABCD*, ***iutA*** **^b^**, *kpsD*, ***kpsM*** **^b^**, *ompA*, ***papC*** **^b^**, *papBDFGIJK*, *senB*, ***sfaBCDEFGXY*** **^b^**, ***vat*** **^a^**, *yagVWXYZ/ecpEDCBA*, *ybtAEPQSTUX*, *ykgK/ecpR*
X9913	12/B2	CTX-M-9, SHV-48, TEM-30	−/+	−	*ant(3″)-Ia*, *qnrS1*	IncN, Col156	*aslA*, ***chuA*** **^a^**, *chuSTUVWXY*, *cnf1*, *csgBDFG*, *entABCDEFS*, *fdeC*, *fepABCDG*, *fes*, *fimABCDEFGHI*, *focH*, ***fyuA*** **^a^**, *gspLM*, *hlyABC*, *iroBCDEN*, *irp12*, *iucABCD*, ***iutA*** **^b^**, *kpsD*, ***kpsM*** **^b^**, *ompA*, *papB*, ***papC*** **^b^**, *papDFGIJK*, *senB*, ***sfaBCDEFGXY*** **^b^**, ***vat*** **^a^**, *yagVWXYZ/ecpEDCBA*, *ybtAEPQSTUX*, *ykgK/ecpR*
X9860	58/B1	CTX-M-1, TEM-1B	+/−	*dfrA5*	*aph(3″)-Ib*, *aph(6)-Id*, *sitABCD*, *sul2*	IncFIB-FII, IncI1, IncQ1	*csgBDFG*, *entABCDEFS*, *espL1R1X145*, *fdeC*, *fepABCDG*, *fes*, *fimABCDEFGHI*, ***fyuA*** **^a^**, *gspCDEFGHIJKLM*, *iroBCDEN*, *irp12*, *iucABCD*, ***iutA*** **^b^**, *ompA*, ***papC*** **^b^**, *papBDEHIJK*, ***sfaX*** **^b^**, *yagVWXYZ/ecpEDCBA*, *ybtAEPQSTUX*, *ykgK/ecpR*
A1197	6756/-	NDM-5, OXA-10	−/−	−	*ant(3″)-Ia*, *aph(3′)-IIa*, *ARR-3*, *cmlA1*, *dfrA14*, *floR*, *OqxA*, *OqxB*, *qnrS1*, *qnrS2*, *rmtB*, *tet(A)*	IncFII, IncX1	*aslA*, *astA*, *csgBDFG*, *entABCDEFS*, *espX145*, *fdeC*, *fepABCDG*, *fes*, *fimABCDEFGHI*, *gspCDEFGHIJKLM*, *ompA*, *yagVWXYZ/ecpEDCBA*, *ykgK/ecpR*

^a^ ExPEC isolates if positive for ≥2 of the following virulence genes (indicated in bold): *papA* and/or *papC* (P fimbriae), *sfa-focDE* (S and F1C fimbriae), *afa-draBC* (Dr-binding adhesins), *iutA* (aerobactin siderophore system), and *kpsMII* (group 2 capsules). ^b^ UPEC isolates if positive for ≥2 of the following genes (indicated in bold): *chuA* (heme uptake), *fyuA* (yersiniabactin siderophore system), *vat* (vacuolating toxin), and *yfcV* (adhesin).

**Table 3:** Genomic profile of the 18 ESBL/CP-*K. pneumoniae* strains submitted to WGS and included in this study.

Strain	ST	ESBL/CP/Other Beta-Lactamase Genes	Class 1 Integron		Non Beta-Lactam Resistance Genes	Plasmid Content	Virulence Profile	KL Type/ wzi
			intI1/3′-CS	Variable Region				
X9872	ST307	CTX-M-15, OXA-1, TEM-1D, SHV-28	+/−	*dfrA14*	*aac(3)-IIa*, *aac(6′)-Ib-cr*, *strA*, *strB*, *qnrB1*, *catB4*, *sul2*, *tet(A)*	IncFIB(K), IncFIB	*ybt*13, ICEKp2, ybST500	KL102/ wzi173
A1188	ST307	CTX-M-15, OXA-48, SHV-28	−/−	−	-	ColpVC, IncL, IncN	−	KL102/ wzi173
A1184	ST307	CTX-M-15, OXA-48 OXA-1, TEM-1D, SHV-28	−/−	−	*aac(6′)-Ib-cr*, *strA*, *strB*, *qnrB1*, *catB4*, *sul2*, *tet(A)*	ColpVC, IncFIB(K), IncL, IncN	−	KL102/ wzi173
A1190	ST307	CTX-M-15, OXA-48, SHV-28	−/−	−	-	ColpVC, IncFIB(K), IncL, IncN	−	KL102/ wzi173
A1192	ST307	CTX-M-15, OXA-48, SHV-28	−/−	−	-	ColpVC, IncFIB(K), IncL, IncN	−	KL102/ wzi173
A1195	ST307	CTX-M-15, OXA-48, SHV-28	−/−	−	-	ColpVC, IncFIB(K), IncHI1-FIB, IncL, IncN	−	KL102/ wzi173
A1196	ST307	OXA-48, SHV-28	−/−	−	-	IncFIB	−	KL102/ wzi173
A1198	ST307	CTX-M-15, OXA-48, SHV-28	−/−	−	*strA*, *strB*, *sul2*	ColpVC, IncFIB(K), IncL, IncN	−	KL102/ wzi173
A1181	ST11	CTX-M-15, OXA-48, OXA-1, TEM-1D, SHV-11	−/−	−	*aac(3)-IIa*, *aac(6′)-Ib-cr*, *strA*, *strB*, *qnrB1*, *catB4*, *sul2*	ColpVC, IncL, IncR	*ybt*10, ICEKp4, ybST112-1LV	KL24/ wzi24
A1182	ST11	CTX-M-15, OXA-48, OXA-1, TEM-1D, SHV-11	−/−	−	*aac(3)-IIa*, *aac(6′)-Ib-cr*, *strA*, *strB*, *qnrB1*, *catB4*, *sul2*	ColpVC, IncL, IncR	*ybt*10, ICEKp4, ybST112-1LV	KL247 wzi24
A1187	ST11	CTX-M-15, OXA-48, OXA-1, TEM-1D, SHV-11	−/−	−	*aac(3)-IIa*, *aac(6′)-Ib-cr*, *strA*, *strB*, *qnrB1*, *catB4*, *sul2*	ColpVC, IncL, IncR	*ybt*10, ICEKp4, ybST112-1LV	KL24/ wzi24
A1194	ST11	OXA-48, SHV-11	−/−	−	-	IncL, IncR	*ybt*10, ICEKp4, ybST112-1LV	KL24/ wzi24
A1183 *	ST15	CTX-M-15, OXA-48, OXA-1, TEM-1D, SHV-28	+/+	*dfrA15-aadA1*	*aac(3)-IIa*, *aac(6′)-Ib-cr*, *strA*, *strB*, *catB4*, *catA1*, *sul2*, *tet(A)*	ColpVC, IncFIB(K), IncHI1, IncL, IncR	−	KL24/ wzi24
A1186	ST15	CTX-M-15, OXA-48, OXA-1, TEM-1D, SHV-28	+/+	*dfrA15-aadA1*	*aac(3)-IIa*, *aac(6′)-Ib-cr*, *strA*, *strB*, *catB4*, *catA1*, *sul2*, *tet(A)*	ColpVC, IncFIB(K), IncHI1B-FIB, IncL, IncR	−	KL24/ wzi24
A1191	ST15	CTX-M-15, OXA-48, OXA-1, TEM-1D, SHV-28	+/+	*dfrA15-aadA1*	*aac(3)-IIa*, *aac(6′)-Ib-cr*, *strA*, *strB*, *catB4*, *catA1*, *sul2*, *tet(A)*	ColpVC, IncFIB(K), IncHI1, IncL, IncR	−	KL24/ wzi24
A1193	ST15	CTX-M-15, OXA-48, OXA-1, TEM-1D, SHV-28	+/+	*dfrA15-aadA1*	*aac(3)-IIa*, *aac(6′)-Ib-cr*, *strA*, *strB*, *catB4*, *catA1*, *sul2*, *tet(A)*	ColpVC, IncFIB(K), IncHI1-FIB, IncL, IncR	−	KL24/ wzi24
A1189	ST326	OXA-48, SHV-28	+/−	*dfrA14*	-	IncFIA-FIB, IncL, IncR	−	KL25/ wzi133
A1185	ST392	OXA-48, OXA-1, TEM-1D, SHV-11	+/−	*dfrA14*	*aac(3)-IIa*, *aac(6′)-Ib-cr*, *strA*, *strB*, *qnrB1*, *catB4*, *sul2*	IncFIB(K), IncFIB, IncL	-	KL27/ wzi187

* mutation in MGRB (colistin resistance).

## Data Availability

The original contributions presented in this study are included in the article/Supplementary Material. Further inquiries can be directed to the corresponding author.

## Supplementary Materials

The following supporting information can be downloaded at: https://www.mdpi.com/article/10.3390/microorganisms13081854/s1, Table S1: Clinical data of ESBL/CP-Ec/Kp collected from 2020–2024; Table S2: Sequencing characteristics for *E. coli* (n = 44) and *K. pneumoniae* (n = 18) clinical isolates analyzed in this study; Table S3: Allelic differences (ADs) between all ESBL/CP-*E. coli* clinical isolates retrieved from 2020–2024; Table S4: Allelic differences (ADs) between ST10/ST58/ST131 ESBL/CP-*E. coli* clinical isolates retrieved from 2020–2024 vs. ESBL-CP/*E. coli* isolates belonging to ST10/ST58/ST131 previosly recovered from storks’ faeces foraged on landfills [30]; Table S5: Allelic differences (ADs) between all ESBL/CP-*K. pneumoniae * clinical isolates retrieved from 2020–2024; Table S6: Allelic differences (ADs) between ST307 ESBL/CP-*K. pneumoniae* clinical isolates retrieved from 2020-2024 vs ESBL-CP/*K. pneumoniae* isolates belonging to ST307 previously recovered fom storks’ faeces foraged on landfills [30].
